# Angiotensin 1 peptide-conjugated CdSe/ZnS quantum dots for cardiac-specific hydrogen sulfide targeted therapy in myocardial ischemia-reperfusion injury

**DOI:** 10.3389/fphar.2024.1435282

**Published:** 2024-10-02

**Authors:** Qing Wang, Xiaofei Xue, Pei Wang, Yue Yu, Jun Wang, Qixia Jiang, Jian Xiao

**Affiliations:** ^1^ Department of Thoracic Surgery, Shanghai Chest Hospital, Shanghai Jiaotong University School of Medicine, Shanghai, China; ^2^ Department of Cardiothoracic Surgery, Changzheng Hospital, Naval Medical University, Shanghai, China; ^3^ Department of Cardiology, Tongren Hospital, Shanghai Jiao Tong University School of Medicine, Shanghai, China

**Keywords:** nanocarrier, quantum dots, cystathionine-γ-lyase, angiotensin 1, endoplasmic reticulum stress, myocardial ischemia/reperfusion injury

## Abstract

**Introduction:**

Myocardial ischemia/reperfusion (I/R) injury remains a major obstacle in cardiovascular therapies. Hydrogen sulfide (H_2_S) shows promise for mitigating I/R injury, but conventional delivery methods, such as NaHS injections or adenovirus-mediated CSE gene therapy, face low efficiency and systemic side effects. This study explores the use of angiotensin 1 (AT1) peptide-conjugated CdSe/ZnS quantum dots (QDs) for targeted delivery of cystathionine-γ-lyase (CSE) plasmids to the myocardium, aiming to boost local H_2_S production and minimize I/R injury.

**Methods:**

CdSe/ZnS QDs were conjugated with AT1 peptides to create a nanocarrier system capable of delivering the CSE plasmid specifically to the myocardium. *In vivo* fluorescence imaging confirmed heart-specific accumulation. Myocardial infarct size, cardiac function, cell death, and oxidative stress were evaluated. Endoplasmic reticulum stress and mitophagy markers, including CHOP/GRP78/eIF2α, were analyzed, and the *CHOP* gene's role was further assessed using an adenovirus vector.

**Results:**

The AT1-conjugated nanocarriers significantly increased CSE expression in the myocardium, as confirmed by fluorescence imaging, without affecting other organs. This localized delivery reduced myocardial infarct size, improved cardiac function, and decreased oxidative stress and cell death. Importantly, a reduction in endoplasmic reticulum stress and mitophagy markers was observed, suggesting that cardioprotection was mediated via the CHOP/GRP78/eIF2α signaling pathway. Reintroduction of *CHOP* using an adenovirus vector reversed these protective effects, confirming the pathway's involvement.

**Discussion:**

This study demonstrates that AT1 peptide-conjugated QDs can effectively deliver CSE plasmids to the heart, providing significant protection against I/R injury through enhanced localized H_2_S production. This approach offers a promising, targeted, and side-effect-free therapy for myocardial I/R injury, with potential for clinical translation.

## Introduction

Recent years have witnessed substantial progress in the prevention and treatment of acute myocardial infarction (AMI). The reperfusion strategy remains the current standard therapy. It includes percutaneous coronary intervention, coronary artery bypass graft surgery, and thrombolytic therapy (Yellon and Hausenloy, 2007). However, myocardial ischemia/reperfusion injury (I/R) limits the efficacy of reperfusion treatment in AMI. I/R acts as a form of reperfusion-induced arrhythmia, myocardial stunning, microvascular obstruction, and lethal myocardial reperfusion injury (Neri et al., 2017). Experimental studies have presented adequate protection for I/R in animal models, but the clinical translations are less satisfying (Binder et al., 2015). The urgent mission for I/R research is to find a translational way for clinical applications (Heusch, 2013).

Hydrogen sulfide (H_2_S) is an endogenous gasotransmitter that plays a vital role in the homeostasis of the cardiovascular system (Citi et al., 2018). Studies have shown that H_2_S has a protective function in I/R (Karwi et al., 2018). Cystathionine-β-synthase (CBS), cystathionine-γ-lyase (CSE/CGL), and 3-mercaptopyruvate sulfurtransferase (3-MST) in mammalian cells produce H_2_S (Shen et al., 2015). The overexpression of cardiac-specific CSE increases the production of endogenous H_2_S, which reduces the infarct size and improves cardiac function (Elrod et al., 2007). Research shows that H_2_S plays a myocardial protective role against IR injury by regulating autophagy via mTOR activation (Xiao et al., 2015). There are H_2_S functions against the apoptosis of cardiomyocytes during IR injury via the miR-1-HDAC4 signaling pathway (Kang et al., 2017). The clinical application of exogenous H_2_S faces many problems. The H_2_S unwanted side effects of systematic delivery include acute change in blood pressure, central neurotoxicity, and respiratory depression (Almeida and Guidotti, 1999).

The H_2_S local delivery or local overexpression of CSE in the heart reduces the side effects but improves efficacy. A nanoparticle or nanocarrier has been developed as a novel delivery system that transports genes, drugs, and other therapeutic agents with cellular targeting ability (Shen et al., 2019). Researchers have combined the nanocarrier and RNA interference (RNAi)-based therapy to enhance cellular uptake, effectiveness, targeted delivery, and limited toxicity (Shen et al., 2019). Angiotensin 1 (AT1) belongs to the renin–angiotensin system (RAS), which plays an essential role in the normal physiology and pathogenesis of cardiovascular diseases (Jiang et al., 2014). The AT1 peptide specifically targets ischemic myocardium due to its high receptor expression in this tissue. By conjugating AT1 to the QDs, we created a cardiac-specific nanocarrier capable of delivering CSE DNA plasmids directly to the heart. This system enhances cellular uptake and gene expression specifically in cardiomyocytes, thereby reducing systemic side effects and improving therapeutic outcomes. A cardiac-specific nanoparticle delivery system containing the CSE DNA plasmid was constructed for the study. The efficiency of the delivery system, the efficacy of the CSE overexpression, the protective effect in I/R, and the underlying mechanism were also evaluated.

## Materials and methods

The synthesis and characterization of CdSe/ZnS quantum dots and oil-soluble CdSe/ZnS core-shell quantum dots (QDs) were purchased from the Suzhou Xingshuo Nanotechnology Company. The QDs were phase-transferred using glutathione (GSH) and cross-linked with carboxyl-PEG-carboxyl groups (Sigma-Aldrich CAT#14565, Mn = 2000) to obtain PEGylated polymer-encapsulated QDs. A measure of 1 mL of the CdSe/ZnS QDs (10 mg) chloroform solution was added to 10 mL of 250 mg GSH plus 200 mg sodium hydroxide aqueous solution, which was magnetically stirred. The chloroform in the mixture gradually evaporated at room temperature. The ligand exchange of QDs is also completed at the water–oil interface. The QD aqueous solution was dialyzed against borate buffer (pH 8.0, 20 mM) for at least 72 h to remove free glutathione from the solution. The glutathione molecules on the QD surface are cross-linked through carboxyl-PEG-carboxyl, which helps improve colloidal stability. The carboxyl group in the PEG crosslinker is activated by the activator 1-(3-dimethylaminopropyl) 3-ethyl carbodiimide hydrochloride (EDC) (10 mg/mL). After 15 min of activation at room temperature, the active PEG crosslinker was added to the GSH-QDs aqueous solution (where the molar ratio of QD/PEG is 1/1000) under magnetic stirring at room temperature for 2 h. The resulting PEGylated QD solution was dialyzed in borate buffer for 72 h to remove unbound PEG and small molecules. Finally, the QD solution was concentrated by rotary evaporation to approximately 5 μM for later use.

### Modification of peptides

Angiotensin 1 (AT1, Gly-Gly-Gly-Gly-Asp-Arg-Val-Tyr-Ile-His-Pro-Phe) is a peptide targeting the ischemic myocardium with lysine residues at the end that was synthesized by GenScript. The purity of the peptide detected by high-pressure liquid chromatography was >95%. The target peptide was covalently coupled to the carboxyl group on the surface of the QDs through the terminal amino group. In the general coupling process, the PEGylated QDs were dispersed in phosphate buffer (pH 6.0, 25 mM), and EDC was added (concentration: approximately 1 mg/mL). The reaction was activated at room temperature for 0.5 h. The excess activator was removed by ultrafiltration, and the targeting peptide (concentration approximately 1 mg/mL) was added and reacted at room temperature for 1 h. The excess targeting peptide was removed using ultrafiltration to obtain the target. QDs were coupled to peptides (peptide-PEG QDs, PPQDs).

### Package of DNA plasmids

Polyvinylimide (Mw 2000) was dispersed in the PB buffer (1 mg/mL). The pH was adjusted to neutral. The targeted PPQDs were mixed with different proportions of polyvinylimide and quickly stirred. Moreover, 1% agarose gel electrophoresis was performed to analyze the electrostatic self-assembly process of QDs and PEI in order to obtain a better QD/PEI ratio. Under a better QD/PEI ratio, the plasmids with different DNA concentrations were mixed with QD/PEI. Agarose gel electrophoresis was used to analyze the plasmids to determine the ratio when loaded onto the QDs (agarose concentration 1%, voltage 80V, and time 15 min).

## Animals

Male SPF-grade Sprague–Dawley (SD) rats (weighing 250–300 g) and c57BL6/J male mice (weighing 18–22 g) were purchased from the Second Military Medical University Experimental Animal Center. The Animal Research Ethics Committee of the Second Military Medical University approved the animal protocols in accordance with the Care and Use of Laboratory Animals published by the US National Institutes of Health (NIH publication vol. 25 no. 28, revised 1996). The experimental animals were kept under standard room conditions with a temperature of 25°C ± 1°C, a humidity of 60%, and natural light from 6 a.m. to 6 p.m. They were routinely given standard rodent chow and water.

The experimental animals were divided into three groups: the sham group, the IR group, and the CSE group. The ischemia/reperfusion (IR) treatment was induced by ligating the left anterior descending artery (LAD) for 30 min, followed by reperfusion for 24 h, as previously described (Xiao et al., 2015). The sham group underwent thoracotomy without LAD ligation. For the IR group, animals were subjected to IR treatment and received an intravenous injection of 20 μL vector nanocarriers immediately before the reperfusion phase. Similarly, animals in the CSE group were subjected to IR treatment and received an intravenous injection of 20 μL CSE nanocarriers immediately before the reperfusion phase. This timing ensured that the nanocarriers were delivered before the reperfusion procedure, allowing for targeted therapeutic intervention during the critical period of reperfusion.

### *In vivo* fluorescence imaging system

The Bruker *in vivo* F PRO system was applied for whole-animal imaging analysis. Images of the post-injection of CSE nanocarriers were captured and analyzed. The excitation and emission filters were set to 410 and 700 nm (bandpass, ±15 nm), respectively.

### Echocardiography

Echocardiography was performed blindly on the mice 24 h after the IR procedure (VisualSonics Vevo 2100, B-mode, and 30 MHz probe). The anesthetized mice were examined at the mid-papillary level. LV dimensions in diastole and systole, especially the ejection fraction (EF), were measured following the standard procedures and calculations.

### Infarct size measurement

Evans blue dye (2%, Solarbio, China) was injected into the vein, but the area at risk (AAR) remained non-dyed (Hu et al., 2020). The heart’s infarct size was assessed using the TTC-staining technique and digital measurement with Image-Pro Plus software (Media Cybernetics). The area at risk/left ventricle ratio (AAR/LV) and scar area/left ventricle ratio were quantified and calculated.

### Apoptosis

TUNEL staining was conducted as previously described (Li et al., 2019a). Image-Pro Plus software (Media Cybernetics) was used to detect the apoptosis rates of TUNEL sections.

### Transmission electron microscopy

The heart tissues from the IR and CSE groups were promptly fixed in 2.5% glutaraldehyde and stored in the same solution at 4°C overnight. Following fixation, the samples were rinsed with phosphate-buffered saline (PBS) and post-fixed in 1% osmium tetroxide for 2 h at room temperature. The fixed tissue was then dehydrated using a graded alcohol series and embedded in an Eponate 12 medium. After these steps—rinsing, fixation, dehydration, and epoxy resin embedding—ultrathin sections were placed on copper grids and stained with lead–uranium double dye. Once cleaned and concentrated, the samples were examined under an electron microscope (FEI-TECNAI-G20) to observe mitochondrial autophagy and captured for analysis.

### Cell culture and treatment

The neonatal cardiomyocytes of rats were cultured and used to conduct the hypoxia-reoxygenation (H/R) experiments ^[9]^. Neonatal rat cardiomyocytes were isolated from 1–3 day old Sprague–Dawley rats using collagenase and trypsin digestion. The cells were then cultured in DMEM with 10% FBS in a humidified atmosphere of 5% CO_2_ and 95% air at 37°C. The cardiomyocytes were divided into four groups:

N + vector group: cultured with 50 μL vector nanocarriers in 5% CO_2_ and 95% air for 24 h.
N + CSE group: cultured with 50 μL CSE nanocarriers in 5% CO_2_ and 95% air for 24 h.
HR + vector group: cultured with 50 μL vector nanocarriers in 1% O_2_, 5% CO_2_, and 94% N_2_ for 24 h and then reoxygenated in 5% CO_2_ and 95% air for 6 h.
HR + CSE group: cultured with 50 μL CSE nanocarriers in 1% O_2_, 5% CO_2_, and 94% N_2_ for 24 h and then reoxygenated in 5% CO_2_ and 95% air for 6 h.
“N” indicates normoxic conditions.

### ATP and LDH test

The conditioned cell culture supernatant (200 μL) was collected after HR and was used to determine LDH levels using a spectrophotometric kit (Roche Diagnostics) and ATP concentrations using an ATP assay (Colorimetric/Fluorometric, Abcam, ab83355) according to the manufacturer’s instructions.

### Western blotting

Protein concentrations were determined using the BCA protein assay kit according to the manufacturer’s protocol. Western blotting (WB) was conducted as previously described. In brief, proteins were separated by SDS-PAGE and transferred to PVDF membranes. The membranes were blocked with 5% non-fat dry milk in Tris-buffered saline with Tween 20 (TBST) for 1 h at room temperature. The membranes were then incubated overnight at 4°C with the following primary antibodies diluted in TBST with 5% bovine serum albumin (BSA): CSE (1:1000, Abcam, ab96755); CHOP (1:1000, Cell Signaling Technology, 2895S); GRP78 (1:1000, Abcam, ab21685); eIF2a (1:1000, Abcam, ab169528); ATF-6 (1:1000, Cell Signaling Technology, #65880); Parkin (1:1000, Cell Signaling Technology, #2132); ATG7 (1:1000, Cell Signaling Technology, #2631); Nix (1:1000, Abcam, ab109414); LC-3 (1:1000, Abcam, ab192890); and GAPDH (1:5000, Thermo Fisher, MA5-15738-D680). After washing with TBST, the membranes were incubated with horseradish peroxidase-conjugated secondary antibodies (1:2000 in TBST with 5% non-fat dry milk) for 1 h at room temperature. The grayscale value of the bands was quantified using Image Lab software (BIO-RAD, United States).

### Statistical analysis

GraphPad Prism 7.0 was used to analyze the data in the study. The continuous data were expressed as the mean ± standard error (SEM). A one-way ANOVA was used, followed by a *post ho*c t Turkey’s test to compare the variables of different groups. A P-value <0.05 was considered statistically significant.

## Results

### Preparation and characterization of peptide-PEG QDs

**FIGURE 1:**
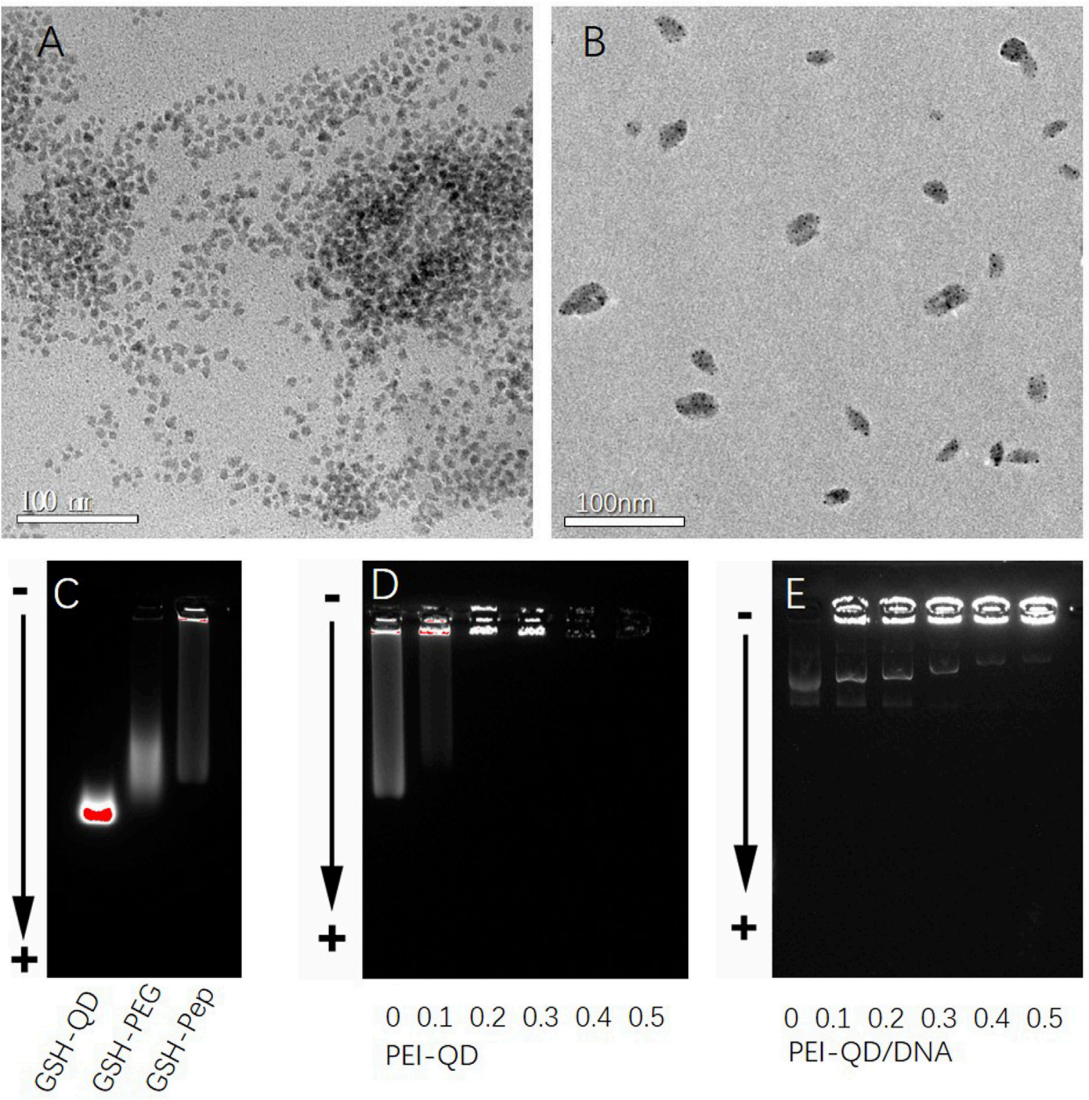
Characterization of different CdSe/ZnS QDs. **(A)** TEM graphs of glutathione-modified CdSe/ZnS QDs. **(B)** TEM graphs of targeted quantum dot clusters loaded with DNA plasmids. **(C)** Agarose gel electrophoresis diagram of QDs with different surface properties from left to right: glutathione, polyethylene glycol PEG, and targeting peptides. **(D)** Agarose gel electrophoresis diagram of different mixed ratios of PEI/QDs. **(E)** Agarose gel electrophoresis diagram after mixing PEI-QDs with different ratios of DNA plasmids. QDs, quantum dots. TEM, transmission electron microscope.

As shown in Figure 1A, the oil-soluble QDs were transferred to the water phase using sulfhydryl-containing glutathione molecules. The QDs modified by the water phase had a good uniform dispersion. The average particle size was approximately 7–10 nm. The surfaces of QDs were modified with PEG to further improve the water solubility of QDs. The carboxyl group on the surface of QDs was covalently coupled with the amino group on the terminal lysine of the targeting peptide through EDC activation. A targeting peptide-modified water-soluble QDs was thus obtained (Figure 1B). As shown in Figure 1C, the agarose gel electrophoresis of QDs with different surface properties showed that the glutathione-modified QDs had the farthest migration distance and no obvious tailing. This indicated that glutathione-modified QDs have a good particle size and charge uniformity. The electrophoretic bands of QDs became more diffuse after PEG modification. The migration distance was reduced. All of this indicated that PEG had been modified on the surface of QDs. However, PEG had some influence on the hydrated particle size distribution of QDs. After further modification of the targeting peptide, the electrophoretic bands of the QDs became wider. This indicated that the targeting peptide can be combined with the PEG-modified QDs. Furthermore, it caused the aggregation of some QDs.

The effect of the PEI/QD ratio on the accumulation of QDs was first studied after obtaining the QDs modified by the targeting peptide. As shown in Figure 1D, when the molar ratio of PEI/QD was 0.2, almost all the QDs were blocked in the electrophoresis loading hole. This demonstrated that the PEI amount at this ratio was sufficient to agglomerate the QDs through electrostatic self-assembly. Finally, a PEI/QD molar ratio of 0.3 was chosen to make PEI a proper excess and prepare DNA carrier clusters. The ratio of PEI-QD/DNA was further studied in order to optimize the embedding conditions of plasmids. The number of DNA plasmids was fixed, and the number of PEI-QDs was adjusted. As shown in Figure 1E, when the molar ratio of PEI-QD/DNA was 0.5, most of the plasmids were wrapped in QD clusters. The successful modification and characterization of PPQDs indicate that the targeted peptide and PEG modifications were effective, resulting in well-dispersed and stable quantum dots. The optimized PEI/QD ratio ensures efficient DNA embedding, crucial for effective gene delivery. This confirms the potential of PPQDs as a viable nanocarrier system for targeted gene delivery.

### PPQD specific enrichment and CSE expression *in vivo*

**FIGURE 2:**
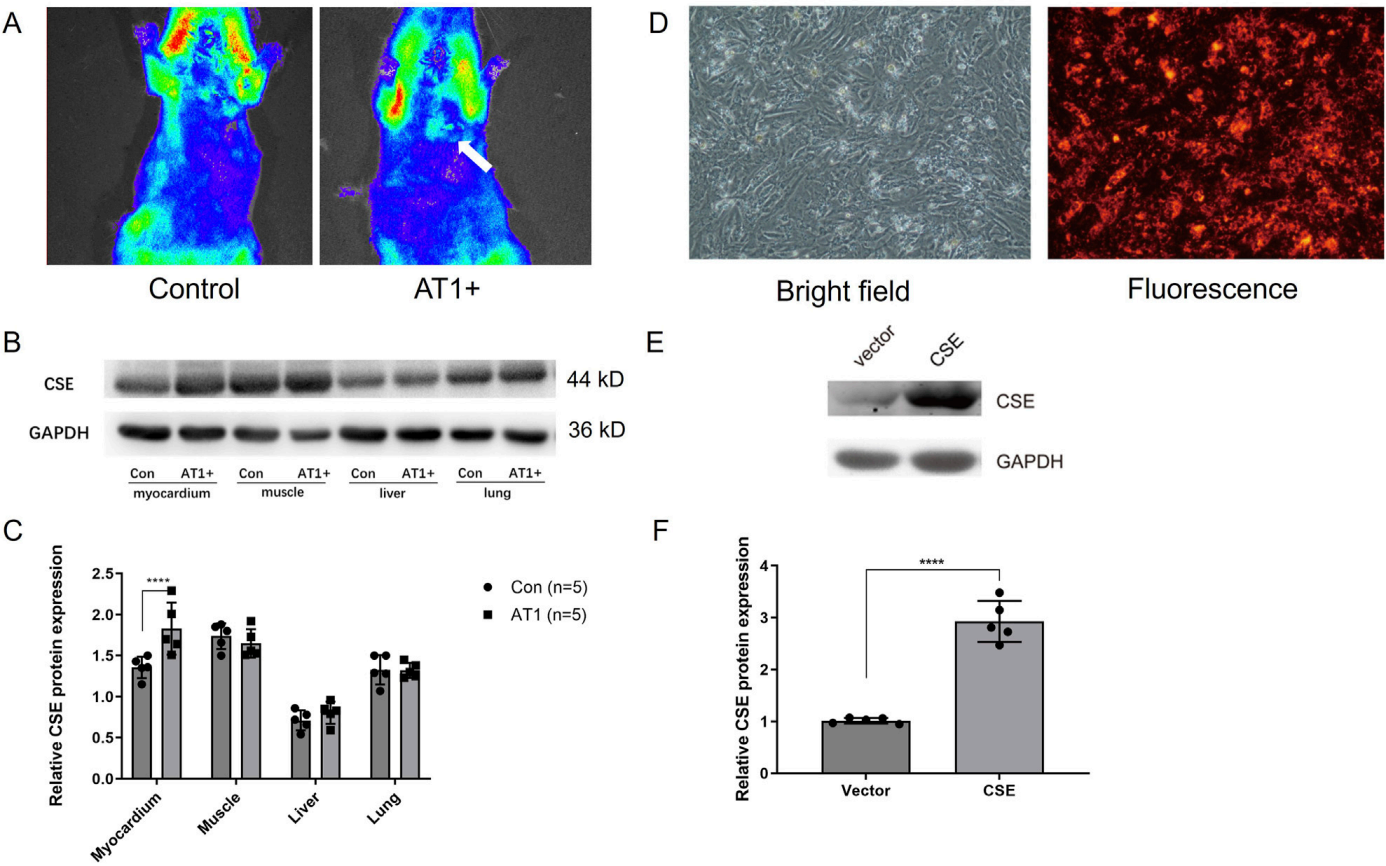
Nanocarrier targeted the myocardium and increased CSE expression *in vivo* and *in vitro*. **(A)** *In vivo* fluorescence imaging system showing nanocarriers conjugated with AT1 enriched in the myocardium of rats. **(B, C)** CSE expression level of different tissues in rats injected with CSE nanocarriers. **(D)** Isolated cardiomyocytes transfected with CSE nanocarriers in bright and fluorescence fields. **(E, F)** CSE expression level in cells transfected with CSE plasmids and the vector. The data are expressed as the mean ± SEM. ****p< 0.0001. CSE, cystathionine-γ-lyase; AT1, angiotensin 1; Con, control group; AT1, AT1 group.

I/R models in rats were established via ligation and release of the left anterior descending (LAD) coronary artery (ligation for 30 min and reperfusion for 24 h). Using nanocarriers’ fluorescence characteristics, the fluorescence imaging system (absorption wavelength of 460 nm) *in vivo* showed that the nanocarrier group’s local myocardium fluorescence carrier AT1 peptide was significantly stronger than that of the empty nanocarrier group (Figure 2A).

CSE nanocarriers with AT1 (AT1 group) and CSE nanocarriers without AT1 (control group) were injected through the internal jugular vein before reperfusion. The rat myocardium, skeletal muscle, and liver and lung tissues were collected. The CSE expression levels in each tissue of IR rats were compared using the Western blot method. The results showed that the expression of CSE protein in the myocardial tissue of the AT1 group (nanocarriers with AT1 and CSE plasmids) was higher than that of the control group. There was no significant difference in the expression of CSE protein between the AT1 and control groups in skeletal muscle and liver and lung tissues. This indicated that the nanocarriers established could target the ischemic myocardium (Figures 2B, C). The findings demonstrate that the AT1-conjugated nanocarriers successfully target the ischemic myocardium, significantly enhancing CSE expression in the heart without affecting other tissues. This specificity is critical for minimizing off-target effects and maximizing therapeutic efficacy, confirming the potential of PPQDs for targeted cardiac gene delivery.

### Transfection of CSE nanocarriers and CSE expression *in vitro*

Neonatal rat cardiomyocytes were isolated and cultured *in vitro*. The standard medium was replaced with 50 μL of CSE nanocarrier (DNA plasmids in PPQDs) medium and observed under a fluorescence microscope. Figure 2D showed that CSE nanocarriers were red fluorescent and widely distributed in cardiomyocytes, indicating that PPQDs containing DNA plasmids have high transfection efficiency for cardiomyocytes. CSE nanomaterials were added to the cardiomyocytes. Thirty hours later, the cardiomyocytes were collected and lysed, while CSE expression was detected using WB. As shown in Figures 2E, F, the CSE nanocarrier group had a significantly higher CSE expression level than the vector group. The *in vitro* transfection results demonstrate that PPQDs can efficiently deliver CSE DNA plasmids into cardiomyocytes, resulting in significantly higher expression of CSE than the controls. This high transfection efficiency and resulting gene expression are crucial for the potential therapeutic application of PPQDs in gene delivery to target cardiac cells.

### Protective effect of CSE nanocarriers for the I/R model *in vivo* and the H/R model *in vitro*

**FIGURE 3:**
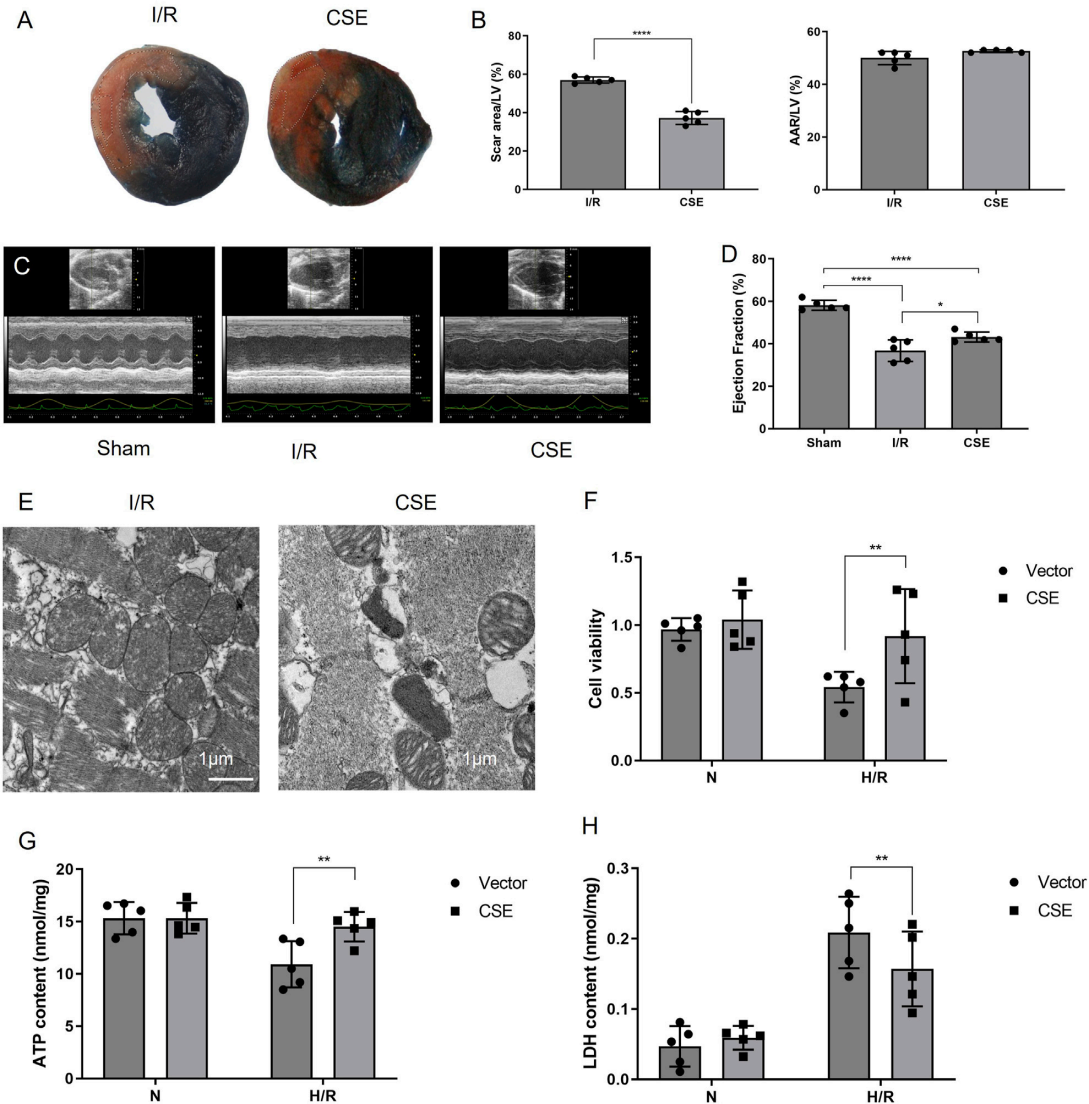
Nanocarriers mitigated I/R and preserved cardiac function. **(A)** TTC/Evans blue staining showing the myocardial infarction area in mice. **(B)** Quantitative indicators of the area at risks/left ventricle and the scar area/ left ventricle. **(C)** Systolic function of different groups detected by echocardiography. **(D)** Ejection fraction of different groups. **(E)** TEM of the myocardium; **(F)** cell viability of cardiomyocytes. **(G)** ATP content of different groups. **(H)** LDH activity of different groups. The data are expressed as mean ± SEM. *p< 0.05, **p< 0.01, and ****p< 0.0001. I/R, myocardial ischemia/reperfusion injury. I/R, IR group treated with IR treatment; CSE, CSE group treated with IR treatment and CSE nanocarriers.

Whether CSE nanocarriers could protect mice from I/R injury *in vivo* was further explored. TTC results showed that the infarct area of mice in the CSE group was lower than that in the control group (*p*< 0.01, Figures 3A, B). This suggested that CSE nanocarriers had a myocardial protective effect *in vivo*. Echocardiography showed that the CSE nanocarriers increased the ejection fraction after I/R (*p*< 0.05, Figures 3C, D). Ultrastructural changes and mitophagy in the myocardium were examined by transmission electron microscopy (TEM). We found that mitochondria decreased more in size, more cristae were lost, and autophagic vacuoles contained more damaged mitochondria in CSE compared to the I/R group (Figure 3E). The abnormal mitochondrial ultrastructure was reversed by the injection of CSE nanocarriers.

**FIGURE 4:**
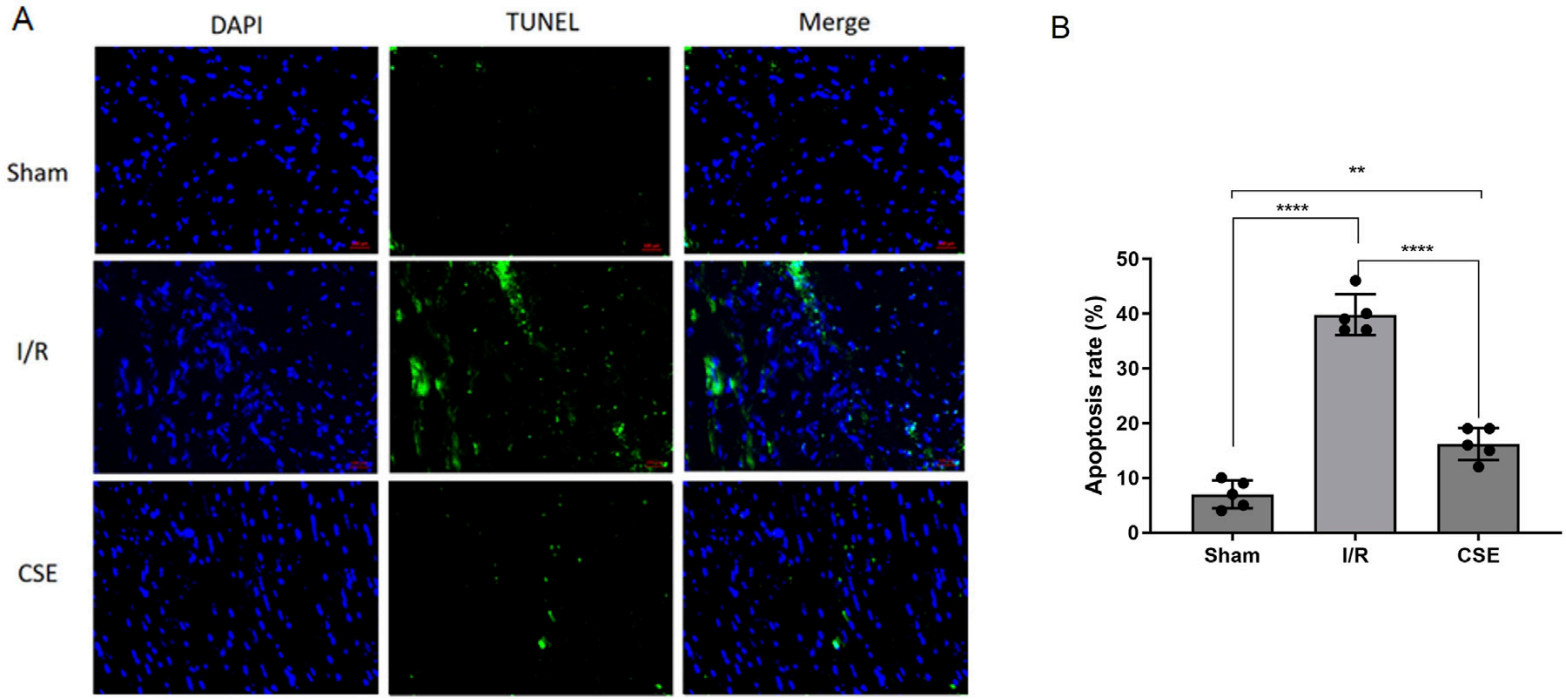
Nanocarrier prevents the apoptosis in the I/R model. **(A)** Representative section of TUNEL staining; **(B)** apoptosis rates of different groups. **p< 0.01; ****p< 0.0001. I/R, myocardial ischemia/reperfusion injury. I/R, IR group treated with IR treatment; CSE, CSE group treated with IR treatment and CSE nanocarriers.

The CCK8 kit was used to detect the viability of cardiomyocytes after H/R, which measures dehydrogenases in the cell. The results showed that the vitality of cardiomyocytes was decreased after H/R injury. However, cardiomyocytes’ vitality in the CSE group was higher than that of the control group. This suggested that CSE nanocarriers could protect the cardiomyocytes *in vitro* (*p*< 0.01, Figure 3F). The ATP detection kit was used to detect the effect of CSE nanocarriers on the ATP content. It indicated that the ATP content of cardiomyocytes decreased after H/R injury. However, the CSE group’s ATP content was higher than that of the control group (*P*< 0.01, Figure 3G). This indicated that CSE nanocarriers had a protective effect on mitochondrial function. The LDH content in the CSE group’s cell culture medium was lower than that of the control group (*p*< 0.01, Figure 3H). As shown in Figure 4, the apoptosis rate of different groups was examined, which showed that apoptosis was more highly inhibited in the CSE group than in the I/R group. The findings from both *in vivo* and *in vitro* models demonstrate that CSE nanocarriers provide significant myocardial protection against I/R injury. They reduce infarct size, improve cardiac function, and enhance cell viability. Additionally, they protect mitochondrial function and inhibit apoptosis. These results underscore the potential of CSE nanocarriers as a therapeutic strategy for mitigating myocardial ischemia-reperfusion injury.

### Inhibition of endoplasmic reticulum stress-mitophagy by CSE nanocarriers in the H/R model

**FIGURE 5:**
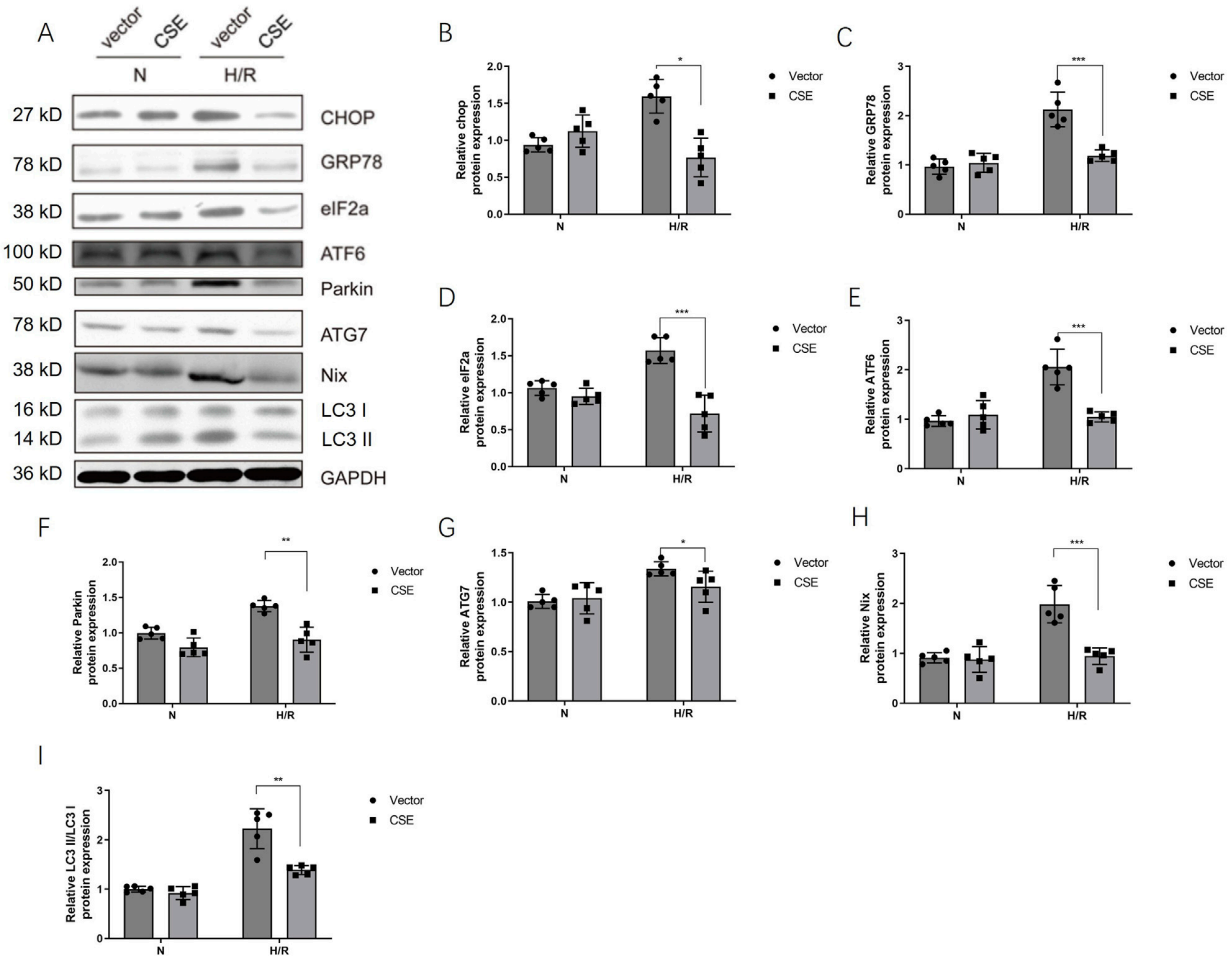
CSE nanocarriers inhibited the protein expression of ERS and mitophagy. **(A)** WB bands of critical markers of ERS and mitophagy. **(B–I)** Quantitative indicators of critical markers of ERS and mitophagy in WB. The data are expressed as mean ± SEM. n = 5 for each group. *p< 0.05, **p< 0.01, and ***p< 0.001. CSE, cystathionine-γ-lyase; ERS, endoplasmic reticulum stress; WB, Western blotting.

Mitophagy and endoplasmic reticulum stress (ERS) play an essential role in myocardial ischemia-reperfusion injury (Zhang et al., 2019). The effect of CSE nanocarriers on mitophagy and ERS was also tested. After adding CSE nanocarriers, ERS-related protein expressions decreased, including CHOP, GRP78, and eIF2a. Furthermore, CSE nanocarriers affected mitophagy-related proteins. CSE nanocarriers inhibited the expression of Parkin, NIX, and ATG proteins in the H/R model. CSE nanocarriers reduced excessive mitophagy and played a role in myocardial protection (Figure 5).

**FIGURE 6:**
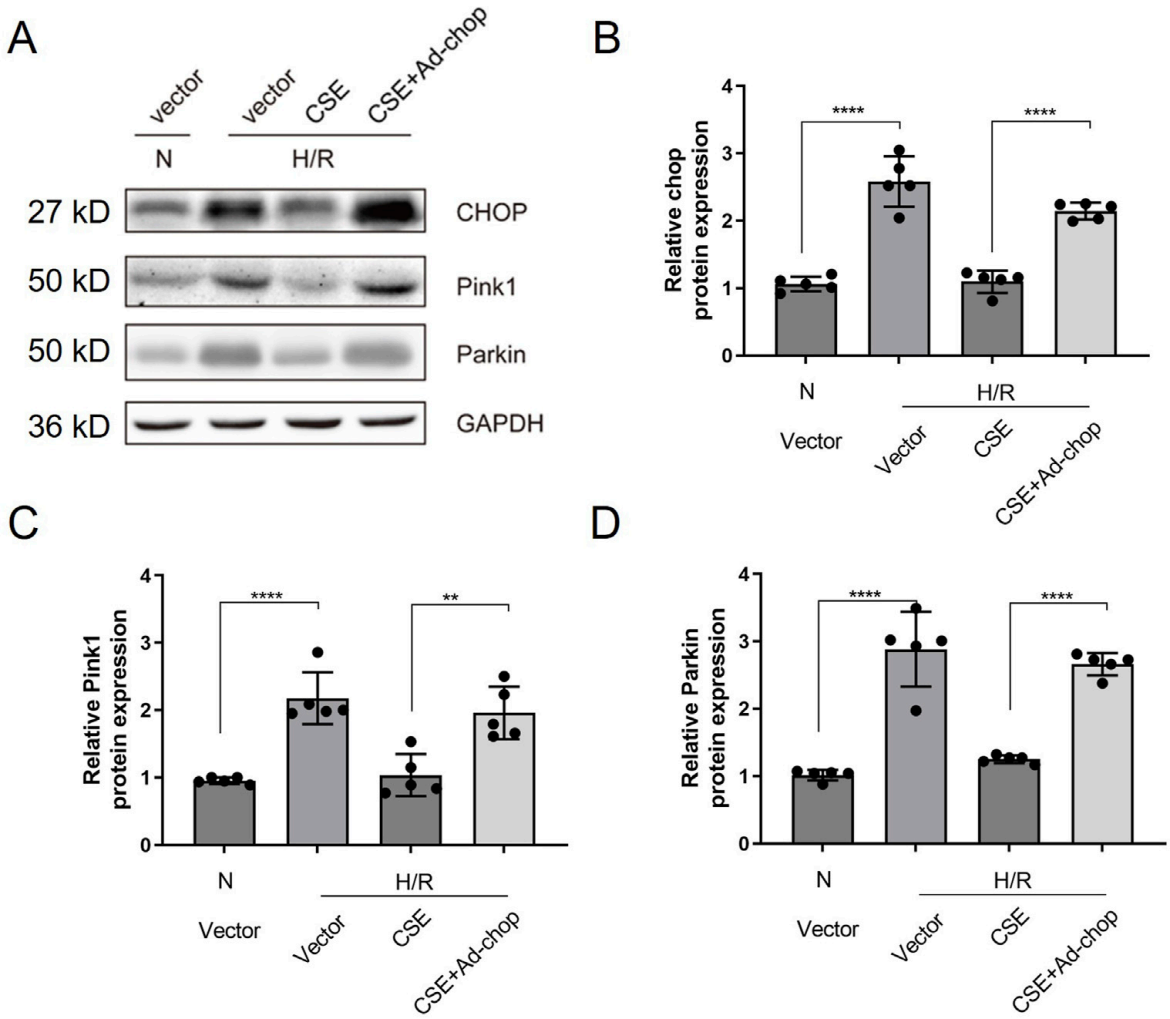
Effect of overexpression of *CHOP* on the inhibition of mitophagy in HR cardiomyocytes by CSE. **(A)** Representative WB bands. **(B–I)** Quantitative indicators of WB results. The data are expressed as mean ± SEM. n = 5 for each group. **p< 0.01; ****p< 0.0001. CSE, cystathionine-γ-lyase; ERS, endoplasmic reticulum stress; WB, Western blotting.

The CHOP adenovirus vector was constructed. The adenovirus carrying the *CHOP* gene was given 24 h before the modeling of the isolated cardiomyocyte HR model. The H/R cell model was divided into four groups: the normal control group, HR group, HR + CSE group, and Ad-CHOP + HR + CSE group. CHOP overexpression attenuated the inhibitory effect of CSE on mitophagy (Figure 6). The results demonstrate that CSE nanocarriers effectively reduce ERS and mitophagy, contributing to myocardial protection in the H/R model. By decreasing the expression of key ERS and mitophagy-related proteins, CSE nanocarriers mitigate cellular stress and damage. This highlights the therapeutic potential of CSE nanocarriers in treating myocardial ischemia-reperfusion injury by targeting ERS and mitophagy pathways.

## Discussion

Nanomedicines, particularly nanoparticles, have seen significant development in clinical applications. Nanocarriers are widely applied in the delivery of genes, drugs, and other therapeutic agents with cellular targeting ability. Nanomedicine gained popularity because of its ability to reduce toxicity by modulating the distribution of drugs and enriching the target sites with chemotherapeutic drugs or recombinant DNA and RNAi molecules (Garbayo et al., 2020). This study developed a nanocarrier system of PPQDs carrying the CSE DNA plasmid, which specifically targeted the heart’s I/R area and demonstrated satisfactory target characterization and protective efficacy.

Different types of nanoparticles have been developed as nanocarriers, including organic and inorganic nanoparticles. As a new inorganic/non-metallic nanoparticle, QDs have attracted adequate attention. This is because QDs have advantages such as good photostability, vigorous fluorescence intensity, and various emission wavelengths (Li et al., 2020). In the study, CdSe/ZnS QDs were chosen as nanocarriers, which is one of the most commonly used QDs. Liu et al. applied CdSe/ZnS QDs to label mesenchymal stem cells (MSCs). CdSe/ZnS-labeled MSCs targeted pancreatic tissues in diabetic rats *in vivo* and significantly reduced the blood glucose levels in diabetic rats (Liu et al., 2015). However, CdSe/ZnS QDs exhibited toxicity in the heart, liver, kidney, immune system, and reproductive system (Li et al., 2019b). CdSe/ZnS QDs are encapsulated with PEG and conjugated with specific peptides to achieve organ-targeted function. Lei and colleagues conjugated the Tat peptide with PEG-encapsulated CdSe/ZnS QDs and introduced them into living mesenchymal stem cells. Characteristic fluorescence of QDs was observed primarily in the liver, lung, and spleen (Lei et al., 2008). Few or no QDs accumulated in the brain, heart, or kidney (Lei et al., 2008). Findings in this study demonstrated that the conjugation of peptides and PPQDs can enrich a nanocarrier in a specific organ. AT1 was used as the target peptide, and we found that the AT1 targets the heart, and AT1 receptor expression was high in the I/R model (Ford et al., 1996). QDs conjugated with targeting peptides were used to transport plasmids to ischemic cardiomyocytes, while their fluorescence allowed tracking of the nanoclusters’ localization. The plasmids were successfully transfected into the cardiomyocytes and increased the protein expression of CSE. The best ratios of the PEI/QD and PEI-QD/DNA were tested in order to achieve a satisfactory transfection efficiency.

The CSE expression was increased in the myocardium without changes in the muscle, liver, and lung after intravenous injection of the nanocarriers containing CSE plasmids. A study has shown that CSE’s overexpression increases the endogenous production of H_2_S, which protects the myocardium (Hu et al., 2020). However, traditional transfection of CSE overexpression in transgenic mice DNA plasmids led to the circulating production of H_2_S and augmented endothelial-dependent vasorelaxation response in the thoracic aorta (Xia et al., 2020). The global effect could possibly cause unwanted side effects. Furthermore, intravenous injection of CSE DNA plasmids had the disadvantage of low transfection efficiency, while local injections in the myocardium were confronted with trauma and low translational values. In this study, the efficacy of the nanocarriers on I/R was further validated. The administration of PPQDs mitigates myocardial injury and preserves the cardiac function. The potential mechanism underlying the protective role in apoptosis, mitochondrial function, and mitophagy was investigated. The ATP content and mitochondrial function of the CSE group were higher than those of the control group. Elrod also studied the potential of H_2_S as a cardioprotective agent. Elrod found that the H_2_S protective effect was associated with the inhibition of myocardial inflammation and the preservation of both the mitochondrial structure and function after I/R (Elrod et al., 2007). The study examined the apoptosis rates of different groups using TUNEL staining. It showed that the CSE group had a decreased apoptosis rate. Previous studies, which investigated the role of apoptosis in I/R, demonstrated that H_2_S protected the cardiomyocytes from IR-induced apoptosis by stimulating Bcl-2 (Li et al., 2019b; Zhang et al., 2019; Kang et al., 2014). Studies have also shown a tight connection and mutual relationship between apoptosis and ERS in I/R (Li et al., 2019b). Li found that dexmedetomidine attenuates I/R in diabetes mellitus by inhibiting ERS (Li et al., 2019b; Zhang et al., 2019).

**FIGURE 7:**
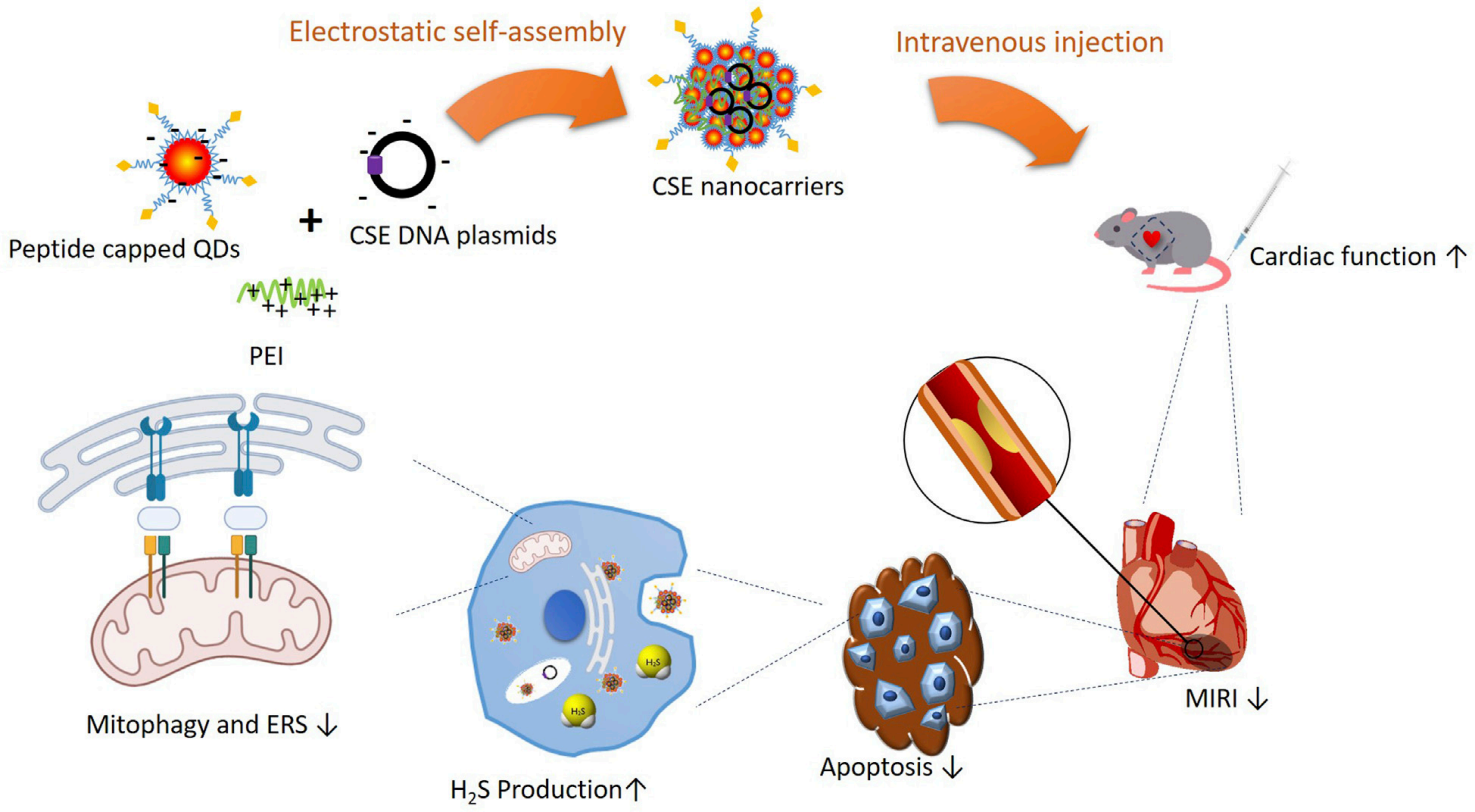
Schematic illustration of PPQDs’ protective effect in I/R and potential signaling pathways.

Furthermore, Zhang and colleagues confirmed that OPA1-related mitochondrial fusion/mitophagy was modulated by melatonin in cardiac I/R injury. The potential roles of mitophagy and ERS in the protective effects of CSE against I/R were investigated. The ERS and mitophagy signaling pathways were activated after the H/R process. However, when administered with nanocarriers carrying CSE plasmids, the signaling pathway was hindered and downregulated. The adenovirus with the *CHOP* gene was applied, which reversed CSE inhibition. The findings stated above supported that CSE nanocarriers protect the heart in I/R, mainly through apoptosis inhibition from ERS and the mitophagy signaling pathway (Figure 7). The promising results in animal models suggest the potential for translating angiotensin 1 peptide-conjugated CdSe/ZnS quantum dots (PPQDs) into human therapies for myocardial ischemia reperfusion injury. However, several critical factors need consideration. First, extensive biocompatibility and safety studies in humans are necessary to ensure long-term safety and minimal adverse reactions. Second, optimizing the dosage is crucial to balancing therapeutic efficacy and toxicity. Third, validating targeting efficiency in human tissues is essential to confirm that PPQDs can specifically target the ischemic myocardium as effectively as in animal models. Additionally, regulatory approval from bodies such as the FDA or EMA requires rigorous preclinical and clinical evaluation to ensure safety and efficacy. The scalability and cost-effectiveness of PPQD production must also be addressed, including developing standardized protocols and ensuring consistency in manufacturing. Finally, comprehensive clinical trials are needed to evaluate the therapeutic benefits of PPQDs in human patients, assessing improvements in cardiac function, infarct size reduction, and overall clinical outcomes. Addressing these factors can facilitate the transition from preclinical studies to human applications, positioning PPQDs as a promising targeted therapy for myocardial I/R injury. This study lays a strong foundation for further investigation and development, highlighting the potential of PPQDs in advancing cardiac therapeutics.

## Conclusion

In summary, a myocardium-specific nanocarrier system was constructed. When carrying the CSE plasmids, PPQDs transfect the cardiomyocytes and increase the CSE expression. Therefore, CSE nanocarriers mitigate the I/R and preserve the cardiac function by reducing apoptosis via the inhibition of ERS and the mitophagy signaling pathway.

## Data Availability

The raw data supporting the conclusions of this article will be made available by the authors, without undue reservation.
